# Prevalence of colonic diverticulosis in mainland China from 2004 to 2014

**DOI:** 10.1038/srep26237

**Published:** 2016-05-17

**Authors:** Wandong Hong, Wujun Geng, Chao Wang, Lemei Dong, Shuang Pan, Xinjing Yang, Maddalena Zippi, Chunfang Xu, Mengtao Zhou, Jingye Pan

**Affiliations:** 1 Department of Gastroenterology and Hepatology, the First Affiliated Hospital of Wenzhou Medical University, Wenzhou, Zhejiang, PR China; 2 Department of Anesthesiology, the First Affiliated Hospital of Wenzhou Medical University, Wenzhou, Zhejiang, PR China; 3 Department of Gastroenterology, the First Affiliated Hospital of Soochow University, Jiangsu, PR China; 4 Unit of Gastroenterology and Digestive Endoscopy, Sandro Pertini Hospital, Rome, Italy; 5 Department of Surgery, the First Affiliated Hospital of Wenzhou Medical University, Wenzhou, Zhejiang, PR China; 6 The First Affiliated Hospital of Wenzhou Medical College, Intensive Care Unit, Zhejiang Province, People’s Republic of China

## Abstract

The aim of this study was to determine the prevalence of colonic diverticulosis in mainland China. Sixty two thousand and thirty-four colonoscopies performed between Jan 2004 and Dec 2014 were reviewed retrospectively. The overall diverticulosis prevalence was 1.97% and out of this, 85.3% was right-sided. Prevalence does not change, significantly, on trends between the period 2004–2014. The peak of prevalence of diverticulosis was compared between the female group aged >70 years to the male one of 41–50 years. The other peak, otherwise, was compared between the group of 51–60 years with the right-sided diverticulosis to the one of >70 years with left-sided disease. Multivariate analysis suggested that the male gender could be a risk factor for diverticulosis in the group aged ≤70 years, but not for the older patients. In addition, among men was registered an increased risk factor for right-sided diverticulosis and, at the same time, a protective one for left-sided localization. In conclusion, the prevalence of colonic diverticulosis is very low in mainland China and it does not change significantly on trends over the time. Both the prevalence of this condition and its distribution changes according to the age and the genders. These findings may lead the researchers to investigate the mechanisms causing this kind of disease and its distribution in regard of the age and the gender.

The prevalence of colonic diverticulosis is thought to be varying across the territories and the ethnics^1^,^2^. Diverticulosis is rare both in Africa and in the developing countries of Asia, but, it’s common in the industrialized areas and in Western^3^. Recent reports suggested that overweight, obesity and physical inactivity are an increased risk for diverticular disease^4^,^5^. As the second largest global economy, China is rapidly undergoing to industrialization and urbanization, resulting in changes of lifestyle and dietary, causing a more fat intake and physical inactivity^6^. The prevalence of diverticulosis it’s known to grow with age, as confirmed by Japanese studies^7^,^8^. China’s aging population is estimated to reach a rate of 5.96 million per year from 2001 to 2020, which is fastly transforming it into an aging nation^9^,^10^. Therefore, there is an hypothesis that the prevalence of colonic diverticulosis could be raised in China over the past decade. However, information about the exact prevalence of colonic diverticulosis in the region of mainland China is limited and outdated in literature^11^.

On the other hand, evolving data suggested that irritable bowel syndrome and colonic diverticular disease may share an underlying pathogenesis, such as micro-biome shifts, visceral hypersensitivity and abnormal motility^3^. Moreover, an overlap between inflammatory bowel disease and diverticular disease has also been noted^3^,^12^. Therefore, a particular interest subsists whether there is a specific gender predilection in diverticulosis, since females show an higher prevalence respect to males as regards both inflammatory bowel disease^13^ and irritable bowel syndrome^14^. Anyway, while reading literature, the relationship between the gender and the presence of diverticulosis is still controversial^8^,^15^.

On the basis of the above mentioned reasons, this study aims to investigate the prevalence and the distribution of colonic diverticulosis in mainland China during the period of the last 11 years and to evaluate the influence of age, gender and yearly trends.

## Results

**Figure 1:**
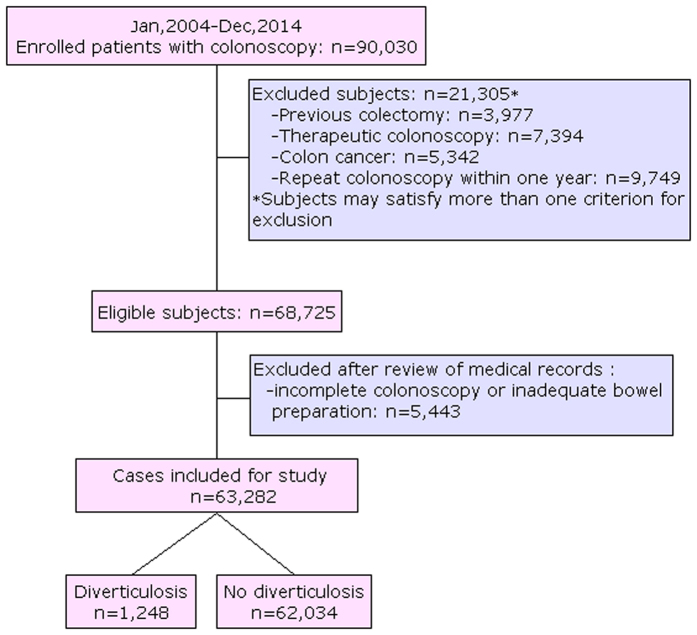
Flow diagram of patients included in the study.

As showed in Fig. 1, a total of 90,030 colonoscopy examinations were performed during 2004–2014 at our hospital. At last, a total of 63,282 (58.13% males) were suitable for inclusion in this study, of which 11,796 (18.64%) were elderly (age groups of 61–70 yrs and >70 years). Overall, 1,248 subjects (76.8% males) had colonic diverticulosis with a prevalence of 1.97% (95% CI: 1.87–2.08%). Patients with diverticula (mean age: 53.0 ± 12.1) were older than those without the disease (mean age: 48.2 ± 13.1) (P < 0.001).

### Age and gender

**Figure 2:**
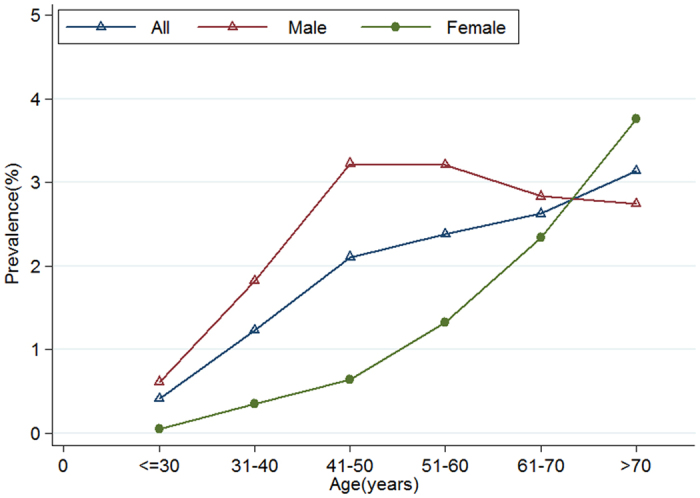
Prevalence of diverticulosis stratified by gender and age.

**Figure 3:**
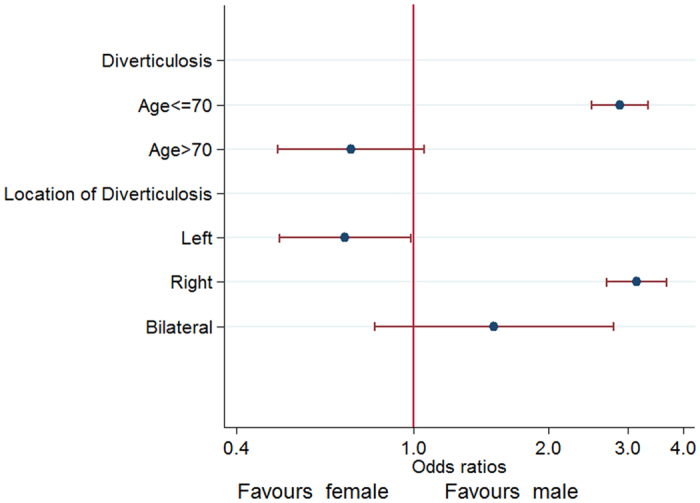
Logistic regression plot of odds ratios and 95% confidence intervals; Effects of gender on the presence of diverticula stratified by age and location of diverticula in colon.

As shown in Fig. 2, the incidence of diverticular disease, in regard to the site, increased rapidly with age. In patients less than 30 years of age, approximately 0.4 percent of them showed an evidence of diverticulosis, while in those ones older than 70 years it was present in 3.1 percent of the cases. For male, the prevalence of diverticulosis increased, rapidly, reaching a peak of 3.22% at the age of 41–50 years and, gradually, in female reaching a peak of 3.76% over 70 years. Prevalence of diverticulosis in male aged ≤70 was always higher than that of female (Fig. 2). Multivariate logistic regression analysis indicated that male gender was a significant risk factor of the presence of diverticulosis for patients aged ≤70 years (OR 2.89; 95% CI 2.50–3.34; P < 0.001) but there were no sex-specific difference in subjects >70 years (OR 0.72; 95% CI 0.50–1.05; P = 0.09), adjusting by age and survey year (Fig. 3).

### Distribution of diverticula

**Figure 4:**
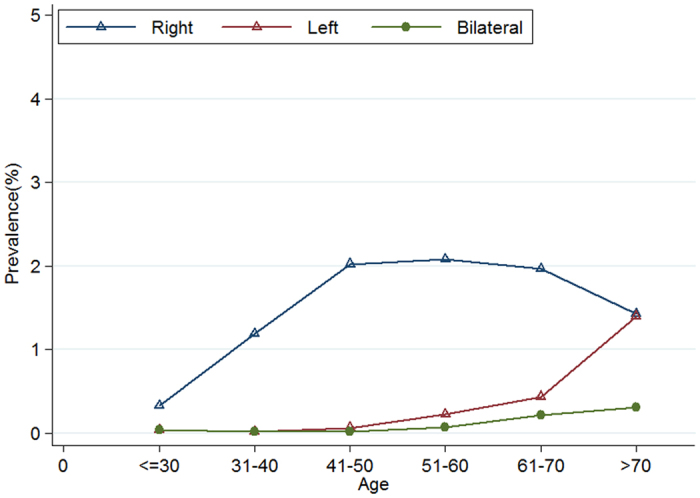
Prevalence of right-sided, left-sided and bilateral diverticulosis according to age.

The distribution of the diverticulosis is shown in Table 1. Out of 1,248 patients, it was right-sided in 85.3% (1065/1248), left-sided in 10.9% (136/1248) and bilateral in 3.8% (47/1248). Patients with right-sided disease (mean age: 51.2 ± 11.1) were younger respect to those ones with left-sided localization (mean age: 64.1 ± 11.8) (P < 0.0001). It was found a greater proportion of males in patients with right-sided disease (859/1065, 80.7%) compared to those ones with left-sided disease (68/136, 50.0%; P < 0.0001). As showed in Fig. 3, the multivariate logistic regression analysis indicated that male was a risk factor for the presence of right-sided diverticulosis (OR 3.15; 95% CI 2.70–3.67; P < 0.001) and was associated to a statistically significant reduction of 30% in the odds for the presence of left-sided diverticulosis compared with female (OR 0.70; 95% CI 0.50–0.98; P = 0.04), adjusting by survey year and age. As shown in Fig. 4, the right-sided diverticulosis prevalence increased rapidly with age and reached a peak of 2.1% in patients at 51–60 years. The prevalence of left-sided diverticulosis, however, only begins to show a marginally rise in patients 51 to 60 years and continues to increase into the seventy decade.

### Yearly trends

**Figure 5:**
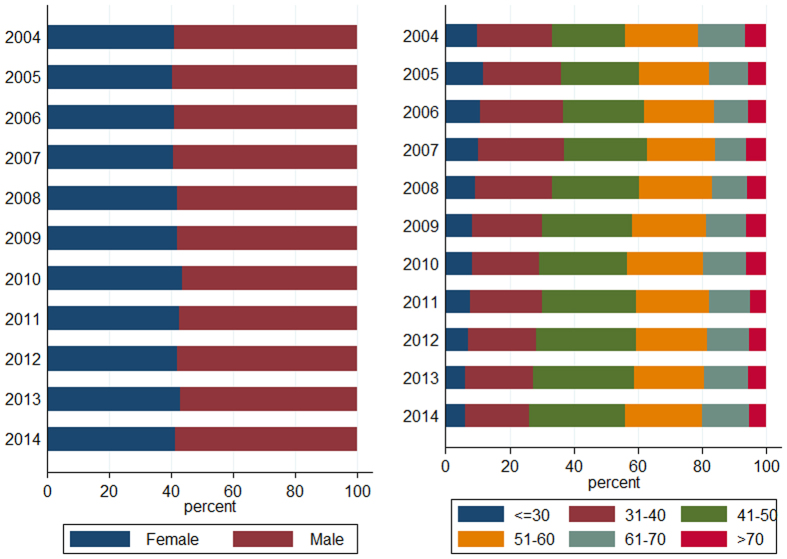
Distribution of gender and age for all patients who underwent colonoscopy from 2004–2014.

**Figure 6:**
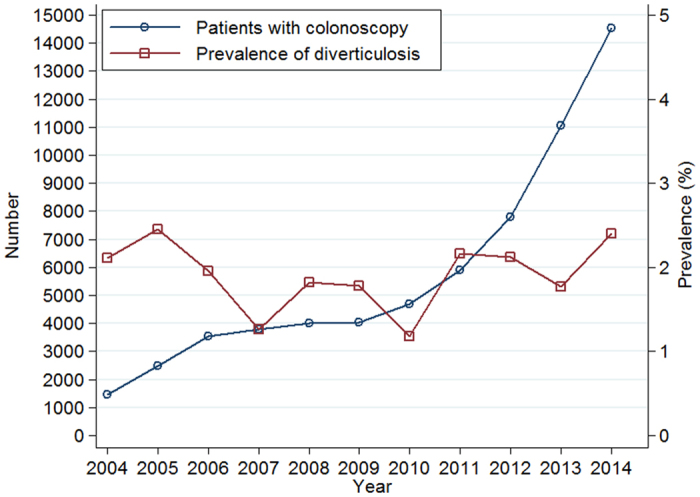
Number of patients who underwent colonoscopy and prevalence of diverticulosis, 2004–2014.

As we can observe in Figs 5 and 6, the proportion of males and elderly patients (age groups of 61–70 and >70 years) did not change significantly, though the overall number of individuals who underwent colonoscopy examinations increased rapidly with survey years (1,468 in 2004 vs. 14,523 in 2014). The prevalence of colonic diverticulosis fluctuated between 2.11% in 2004 and 2.40% in 2014, but not significantly changed with survey year (Fig. 6). Multivariate logistical regression revealed that the survey year was not associated to the presence of diverticulosis, adjusting by gender and age (OR 1.02; 95% CI 1.00–1.04; P = 0.10).

## Discussion

The prevalence of diverticulosis varies worldwide depending on different populations. The overall prevalence of this condition in our study was 1.97%, comparable to the result (1.2%) reported by Pan *et al*.^11^ from China three decades ago. At the same time, however, it was significantly lower respect the results highlighted in recent reports, coming from our neighboring nations, such as Thailand (28.5%)^16^, Japan (20.0–25.8%)^7^,^8^,^17^ and Singapore (45.0%)^1^. This difference may be, mainly, attributed to different race, genetic predisposition^18^, dietary habits and lifestyle^19^. Peery *et al*.^20^ found that non-white participants showed a 26% lower risk of diverticulosis towards whites, suggesting how race was a risk factor independent from diet, smoking and other lifestyle factors. Strate *et al*.^21^ confirmed that genetic factors contribute to diverticular disease susceptibility in a population-based study of twins and siblings. Reichert *et al*.^18^ suggested that diverticulosis should be considered as a complex genetic disease resulting from environmental factors interacting with multiple susceptible genes and disease modifiers. In addition, the proportion of the elderly patients (age group of 61–70 yrs and >70 years), who underwent colonoscopy examination, only 18.64% resulted to be lower compared to other studies (49.2% 1–68.5% 7). This may also contribute to the low overall prevalence of diverticulosis in our current data due to the fact that diverticulosis is age-dependent. The Chinese tradition of taking care of old people is now threatened by urbanization, once child policy, emigration^10^, as well as stagnation in the development of geriatrics and inadequate medical resources^9^ may be explanations of low proportion of elderly individuals in our study. Therefore, the program of promoting the development of geriatric medicine still has a long way to be taken in China.

Diverticulosis is thought to develop from age-related degeneration of the mucosal wall and segmental increases in colon pressure, resulting in bulging through the points of weakness^3^. As expected, when men and women are combined, our data showed how the prevalence of diverticulosis increases with the age, which was in keeping with other studies^8^,^17^,^22^ (Fig. 2).

In regard to the distribution of diverticula, our data underline how the 85.3% of the cases of diverticulosis were located in the right side of the colon. This point of view is in accord with previous observations demonstrating that the anatomic distribution pattern of diverticulosis is, predominantly, left-sided in the West and right-sided in the Asia^7^,^15^,^23^.

Patients with right-sided disease were younger than the ones left-sided(P < 0.0001)(Table 1).This result is consistent with the previous reports^23^. As shown in Fig. 4, the prevalence of right-sided diverticulosis rapidly increased with age and reached a peak of 2.1% in patients at 51–60 years of age. The prevalence of left-sided diverticulosis, however, only begins to show a marginally rise in patients aged 51 to 60 years and continues to increase to a peak at the age above the 70 years. Similar results was also confirmed by Fong *et al*.^1^, which have observed how the right diverticular disease does not continue to increase in frequency in the elderly aged >60 with aging, while the prevalence of left diverticular disease increase into the eighth decade. Japanese researchers have noted that the presence of left-sided diverticulosis was associated to an higher risk of irritable bowel syndrome^24^.These results also suggested that the pathogenesis of right-sided diverticulosis may be different from left-sided disease^25^. While most data of colonic diverticulosis have been collected by Western patients, in whom left-sided diverticulosis predominates, the pathophysiology of right-sided diverticulosis remains unclear^26^. It was thought that the majority of the right sided diverticulosis might be self-limiting^23^ and congenital^16^,^17^. Left sided diverticulosis is thought to be acquired, as the result of low fiber diet and changes in colonic motility and in the connective tissue of the colonic wall^27^.

Data on the association between gender and the presence of diverticulosis is somewhat conflicting. Most studies found that there are no gender-specific predilection for diverticulosis^1^,^15^,^17^,^28^. However, our study, as well as two recent large cohort studies^7^,^8^ showed that different gender displays distinct prevalence rates. The prevalence of diverticulosis steadily increased with age and reached a peak of 3.76% in female aged >70 years while it reached a peak of 3.22% in male aged 41–50 years (Fig. 2). Multivariate analysis suggests that male could be a risk factor for diverticulosis in patients aged ≤70 years (OR 2.89; 95% CI 2.50–3.34), but not for patients aged >70 years (Figs 2 and 3).

The relationship between gender and distribution of diverticula is poorly investigated. Out data showed a greater proportion of males in patients with right-sided diseases (80.7%) compared to those ones with left-sided diseases (50.0%; P < 0.0001). As showed in Fig. 3, multivariate analysis indicated that male represents a risk factor for the presence of right-sided diverticulosis (OR 3.15; 95% CI 2.70–3.67), but was associated with a statistically significant 30% reduction in the odds of presence of left-sided diverticulosis when compared with female (OR 0.70; 95% CI 0.50–0.98), adjusting by survey year and age. Nagata *et al*.^7^ reported that male was a risk factor for right-sided and bilateral diverticula, but not finding association between gender and left-sided diverticula. The discrepancies between our data and the findings of Nagata *et al*.^7^ need further investigation. These may be partly attributed to the difference in the sample size and in the inclusion criteria. The way in which the gender contributes to the pathogenesis of the diverticulosis is unclear, thought it is now becoming widely recognized that there are important sex differences in many disease^29^. A growing body of evidence shows that there are some sex-associated differences in gut community composition and metabolic activity^30^. In addition, Sankaran-Walters *et al*.^31^ suggested an up-regulation in gene expression related-immune functions in the gut microenvironment of women compared to men, in the absence of disease or pathology. Moreover, sex differences in the mucosal immune system may predispose women to inflammation-associated diseases that are exacerbated following menopause^31^. At last, Ober *et al*.^32^ and Morrow *et al*.^29^ suggested that sex-specific genetic architecture also plays a role in contributing to quantitative traits and disease risk in the contemporary humane populations apart from classical differences in circulating hormones.

Most of the studies have reported an increase in the prevalence of diverticulosis over the last two decades due to the coming of aging society and the adoption of a western dietary intake and lifestyle. China counts a population of over 1.3 billion people of which 160 million are age 60 and older, representing the largest aged population in the world^9^. However, contrary to our expectations, the prevalence of colonic diverticulosis does not significantly change with survey year (Fig. 6). Multivariate analysis also indicated that the survey year was not associated with the presence of diverticulosis adjusting by gender and age (OR 1.02; 95% CI 1.00–1.04). In our study, this differences may be partly explained by the fact that the proportion of gender and elderly (age groups of 61–70 and >70 years) of individuals who underwent colonoscopy examinations did not significantly changed with survey year (Fig. 5). On the other hand, this may also suggest that racial and genetic predisposition may have a stronger impact on the development of colonic diverticulosis than dietary habits and lifestyle in Chinese population.

The strength points of this study include a large sample size that gives the study enough statistical power and all diverticulosis are diagnosed by endoscopy which may reduce the study heterogeneity. To our best knowledge, this is the first study to investigating the prevalence of diverticulosis in Chinese population stratified by age, gender and survey year in mainland China. There are also some limitations in the present study, mainly due to the retrospective analysis. Firstly, elderly people prevalence is too low in the studied population, which could influence the final analysis, since our study found left-sided diverticulosis mainly in the older population. Therefore, it would be appropriate to interpret these findings with caution and, subsequently, validate these results with a prospective study on a large scale. Another point of interest is that, generally, dietary fiber has been considered as the major protective factor for the developing of diverticulosis^33^,^34^,^35^. This kind of diet aims to normalize colon motor activity^19^, increase stool transit time and alter the bacteria in the gut^34^. As people often take the fibers from a variety of foods, some studies^34^,^36^ also investigated the association between the type of dietary fiber and diverticular disease. A prospective study^36^ suggested that the insoluble fibers were significantly associated with a decreased risk of diverticular disease. Recently, Crowe *et al*.^34^ confirmed that the relative risk for diverticular disease occurance was significantly reduced with an increasing intakes of fibers from cereals and fruit, but not for the ones from vegetables or potatoes. Regrettably, detailed dietary was not recorded in current study, due to the retrospective study design. Evidence from literature indicated that dietary fiber consumption among Chinese adults aged 18–45 years decreased from 1989 (22.6 g/day) to 2006 (17.8 g/day^37^), while the trends in the average total dietary fiber intake in Chinese adults aged 45 years and above(19.0 g/day^38^) remained at a stable level in the past decade in mainland China. In addition, the average daily total dietary fiber intake among Chinese adults aged 45 years and above was higher than that of Western countries such as United States (15.9 g/day^39^) and France (16.0g/day^40^). On the other hand, the main dietary pattern of our region is a traditional southern dietary pattern, characterized by high intakes of rice, fresh leafy vegetables, low-fat red meat, pork, organ meats, poultry and fish/seafood and low intakes of wheat flour and maize/coarse grains^41^,^42^. In addition, this dietary pattern in Chinese adults from 1991 to 2009 remained stable over time, meaning that the choose of people to combine their foods remained relatively stable, despite rapid economic changes in China and rapid increase in dietary diversity^42^. It has been suggested that the traditional southern dietary pattern was associated to a lower risk of hypertension^41^, stroke^43^ and diabetes^44^. As a result, it is assumed that feature of dietary habits or fiber intake of our population may at least partly contribute to the low prevalence of diverticulosis and its yearly trends in present study. It would be necessary and interesting to investigate relationship between fiber intake, dietary pattern and colonic diverticulosis in mainland China in the future. Finally, the actual prevalence of colonic diverticulosis is difficult to determine, because most people with colonic diverticula are asymptomatic and may not present for colonic evaluation. At last, our patients were from a single center in a medium-sized City of China that might not be representative of the entire Chinese population in mainland China, since lifestyle and dietary habits vary from different regions in China^6^.

In conclusion, the prevalence of colonic diverticulosis is very low in mainland China and it does not change significantly on trends from 2004 to 2014. The prevalence of diverticulosis and its distribution changes with age and between genders. Except racial, genetic and environment factors, this knowledge may guide researcher to investigate disease-causing mechanisms of diverticulosis and its distribution depending on age and gender.

## Materials and Methods

### Inclusion and exclusion criteria

Eligible for the study were patients who underwent colonoscopy examination in the First Affiliated Hospital of Wenzhou Medical University, between Jan 2004 and Dec 2014. Exclusion criteria included: therapeutic colonoscopy, colon cancer, prior colonic resection, incomplete examination or inadequate bowel preparation and repeated colonoscopy within one year. This study protocol was approved by the Ethics Committee of the First Affiliated Hospital of Wenzhou Medical University.

### Data collection and definition

The definition of complete colonoscopy and the classification of bowel preparation was described by Ashktorab *et al*.^45^. Diverticulosis was defined as the presence of colonic diverticula irrespective if these are clinically silent, symptomatic or complicated^3^. Gender, age and distribution of diverticulosis were recorded. Age was divided into a categorical variable consisting of six groups as follows: ≤30, 31 to 40, 41 to 50, 51 to 60, 61 to 70 and >70 years olds. As described by Yamada *et al*.^26^, colonic diverticulosis were shared by location into right (cecum, ascending colon and transverse colon), left (descending colon, sigmoid colon and rectum) and bilateral (right, transverse and left sections of the colon) sides of the colon.

### Statistical analysis

Continuous values were expressed by mean ± SD and compared using the independent-samples t-test. Categorical values were described by count and proportions and compared by the χ2 test. A multivariate logistic regression analysis was used to evaluate the associations between the prevalence of diverticulosis and sex, age category and survey year of patients. Odds ratios (OR) were calculated with 95%CI. Two-sided P-values ≤0.05 were considered statistically significant.

## Additional Information

**How to cite this article**: Hong, W. *et al*. Prevalence of colonic diverticulosis in mainland China from 2004 to 2014. *Sci. Rep.* **6**, 26237; doi: 10.1038/srep26237 (2016).

## Figures and Tables

**Table 1:** Distribution of diverticulosis by gender, age group (n = 1248).

**Variable**	**Left**	**Right**	**Bilateral**
Gender (number)			
Women	68	206	15
Men	68	859	32
Age group (number)			
≤30	2	16	2
31–40	3	165	3
41–50	12	371	3
51–60	33	300	10
61–70	36	162	18
>70	50	51	11
Mean age (years)	64.1 ± 11.8	51.2 ± 11.1	60.9 ± 13.5
